# QSAR Models for Predicting Five Levels of Cellular Accumulation of Lysosomotropic Macrocycles

**DOI:** 10.3390/ijms20235938

**Published:** 2019-11-26

**Authors:** Ulf Norinder, Vesna Munic Kos

**Affiliations:** 1 Swetox, Karolinska Institutet, Unit of Toxicology Sciences, Forskargatan 20, SE-151 36 Södertälje, Sweden; ulfn@dsv.su.se; 2 Department of Computer and Systems Sciences, Stockholm University, Box 7003, SE-164 07 Kista, Sweden; 3 Department of Physiology and Pharmacology, Karolinska Institutet, SE-171 77 Stockholm, Sweden

**Keywords:** cellular accumulation, macrocycle, QSAR, OPLS, applicability domain, classification models, molecular descriptors

## Abstract

Drugs that accumulate in lysosomes reach very high tissue concentrations, which is evident in the high volume of distribution and often lower clearance of these compounds. Such a pharmacokinetic profile is beneficial for indications where high tissue penetration and a less frequent dosing regime is required. Here, we show how the level of lysosomotropic accumulation in cells can be predicted solely from molecular structure. To develop quantitative structure–activity relationship (QSAR) models, we used cellular accumulation data for 69 lysosomotropic macrocycles, the pharmaceutical class for which this type of prediction model is extremely valuable due to the importance of cellular accumulation for their anti-infective and anti-inflammatory applications as well as due to the fact that they are extremely difficult to model by computational methods because of their large size (M_w_ > 500). For the first time, we show that five levels of intracellular lysosomotropic accumulation (as measured by liquid chromatography coupled to tandem mass spectrometry—LC-MS/MS), from low/no to extremely high, can be predicted with 60% balanced accuracy solely from the compound’s structure. Although largely built on macrocycles, the eight non-macrocyclic compounds that were added to the set were found to be well incorporated by the models, indicating their possible broader application. By uncovering the link between the molecular structure and cellular accumulation as the key process in tissue distribution of lysosomotropic compounds, these models are applicable for directing the drug discovery process and prioritizing the compounds for synthesis with fine-tuned accumulation properties, according to the desired pharmacokinetic profile.

## 1. Introduction

The pharmacokinetic profile is of critical importance for a drug’s success, and models to predict absorption, distribution, metabolism and excretion (ADME) properties are an extremely valuable tool in drug discovery. Among the ADME processes, in vitro and in silico modeling are most often applied to optimize the drug’s absorption and interaction with metabolic enzymes. For some types of drugs, however, their distribution in the organism and retention in tissues can markedly vary with structure, and dramatically affect the overall pharmacokinetic profile. Being able to predict these properties for new compounds before synthesizing them can be a huge advantage in the drug discovery phase.

Lysosomotropic drugs reach very high concentrations in cells and tissues, resulting in a pharmacokinetic profile characterized by a very high volume of distribution, very low plasma concentrations, and often long halftimes in tissues [1]. Due to their cationic and amphiphilic nature, lysosomotropic compounds accumulate in acidic compartments of the cell such as lysosomes, based on the proton trapping mechanism [2,3]. Briefly, the neutral form of the compound passes through the cell membrane and subsequently through the lysosomal membrane. The compound becomes protonated in an acidic environment, and as such, cannot traverse back through the membrane. Consequently, the equilibrium of the drug movement in the cell is markedly shifted toward movement into the lysosome, resulting in very high overall intracellular concentrations. 

Such a pharmacokinetic profile is considered beneficial for drugs that need high tissue penetration such as macrocycle antibiotics (e.g., erythromycin, clarithromycin, and azithromycin), enabling them a long-lasting action on intracellular pathogens [4] as well as being essential for their anti-inflammatory activity [5]. In addition, a less frequent dosing regime, suitable for such compounds due to their high concentration in tissues, contributes to a better patient compliance. Thus, finding a compound with optimal tissue distribution for a given application offers a significant advantage over compounds with a more classical pharmacokinetic profile.

Apart from providing pharmacokinetic benefits, lysosomotropic accumulation affects cells. For moderately accumulated compounds, accumulation in lysosomes results in an increase in lysosomal volume, and at higher intracellular accumulation, accumulation of phospholipids is observed, both phenomena proven reversible after removal of the drug [6,7]. Extremely highly accumulating compounds can, however in some cell types, cause cell death. 

Therefore, to be able to utilize the benefits of intracellular accumulation and at the same time control potential negative effects, it is extremely important to understand the structural features governing the accumulation of lysosomotropic compounds.

So far, in silico modeling of lysosomotropic compounds, or cationic amphiphilic drugs (CADs), has largely revolved around the prediction of phospholipidosis as a clinically detectable adverse effect of extreme compound accumulation, rather than the prediction of their intracellular concentration, which would help fine tune the ADME properties. For small molecules, a few in silico models exist for the prediction of CADs or phospholipidosis inducers. These, however, usually classify molecules in only two classes, based on whether they are likely or not to be an inducer, hence a lysosomotropic compound. These in silico models, however, are unable to predict macrocycles correctly due to the large molecular size (M_w_ > 500) and complex 3D structure [8]. Similarly, current physiology based pharmacokinetic (PBPK) models developed for small molecules often struggle to describe the disposition of macrocyclic drugs, partially due to tissue partitioning models used within [9,10], which are not able to capture their true distribution properties and the level of accumulation in tissues. A new set of rules (“Beyond Rule of 5”) is needed to better describe successful macrocycle pharmaceuticals [11,12], but this is limited by the scarcity of data and systematic knowledge on the relationship between macrocycle structure and their pharmacokinetics [13]. Therefore, the preclinical assessment of the pharmacokinetic profile of highly accumulating macrocycles is largely limited to costly animal studies.

Considering a highly complicated synthesis procedures for macrocyclic compounds, often with more than 15 steps and very low yield [14], having a reliable model that could predict accumulation in cells solely from molecular structure, without any experimental data, would be of great help in the early drug discovery phase for the prioritization of macrocyclic compounds with optimal pharmacokinetic properties for synthesis. In addition, models capable of giving a more refined answer than just yes or no for lysosomotropic compounds and differentiate various levels of accumulation intensity, could markedly improve current predictions of pharmacokinetic properties for a broader range of compounds. 

In quantitative structure–activity relationship (QSAR) models, multiple molecular descriptors (theoretical, calculated, or experimental) of a set of molecules are quantitatively assessed for their contribution to the measured biological activity, resulting in a model capable of predicting the level of the biological activity for new compounds based on the values of the descriptors found to contribute most to the given activity. These models are a valuable in silico tools in drug discovery to direct the synthesis of new compounds with an increased likelihood of producing desired pharmacological activity for which the QSAR model is available. 

Our first in silico models to predict macrocycle intracellular accumulation were 2-class decision tree models, which, apart from calculated molecular descriptors, relied on experimentally determined physicochemical properties such as lipophilicity parameter ChromlogD, or phospholipophilicity parameter CHIIAM [15]. Our later studies investigated the applicability of calculated 3D molecular descriptors with or without empirically determined lipophilicity to develop continuous prediction models for cellular accumulation [16]. Recently, we have developed a high throughput in vitro model for the indirect measurement of the cellular accumulation of compounds [17]. We have shown that by imaging the increase in lysosomal volume, we can, with 81% accuracy, predict five levels of accumulation intensity for existing compounds, which is sufficient to successfully rank the substances available for testing, according to this pharmacokinetic property. 

In the present study, we go one step further, and on a broader set of compounds, use the data available from lysosomal imaging to develop advanced, purely in silico descriptor-based models to enable the prediction of the level of cellular accumulation for compounds without the need for experimental testing. First, we developed a prediction model for intracellular accumulation by combining the calculated molecular descriptors and experimental data on the imaging of the lysosomal volume increase. Then, we developed an additional model that was able to predict the experimental imaging data. By fusing the information from the two models, we obtained a novel in silico model capable of predicting five levels of intracellular accumulation solely from the molecular structure with 60% balanced accuracy (i.e., 3-fold improvement from baseline random distribution).

## 2. Results and Discussion

### 2.1. Experimental Determination of Intracellular Accumulation

So far, there are two ways to experimentally determine the intracellular accumulation of lysosomotropic compounds in cells in vitro:

1. Quantification of compound concentration in cells by liquid chromatography coupled to tandem mass spectrometry (LC-MS/MS), which is a low throughput method, that is often expensive, requires larger sample, and is rather time consuming to adjust reliable and robust measurements. On the other hand, when validated, the method gives absolute quantification of accumulation (ACC), which can subsequently be used to divide compounds in different levels (classes), according to the intensity of accumulation (here denoted as ACC Class (Table 1)).
2. Indirect evaluation of compound accumulation from the measured increase in lysosomal volume induced by the accumulating compound, performed by fluorescent cell microscopy (cell imaging). This method is high throughput, but less precise than the LC-MS/MS method, and can only give an estimation of the accumulation classified in five classes (denoted as LTR ACC Class). Still, this imaging method has an accuracy of 81% in predicting the actual ACC Class (measured by LC-MS/MS) [17].

In the present study, we used 69 macrocycles and eight non-macrocycles with measured intracellular accumulation by the LC-MS/MS method and classified in five classes (ACC Class), out of which 39 macrocycles and all eight non-macrocycles also had data on the indirect evaluation of accumulation by cell imaging (LTR ACC Class) (Table 1). We then used 97 calculated physicochemical molecular descriptors, together with the experimental LTR ACC Class data, to develop orthogonal projections to latent structures (OPLS) models for the prediction of actual intracellular accumulation (i.e., parameter ACC Class). The calculated descriptors cover molecular size and shapes, charges, ring characteristics, lipophilicity, and hydrogen bonding as well as various van der Waals surface area contributions of hydrophobic/hydrophilic, polarizability, and electrostatic nature (listed in the Supplementary Materials). 

**Figure 1:**
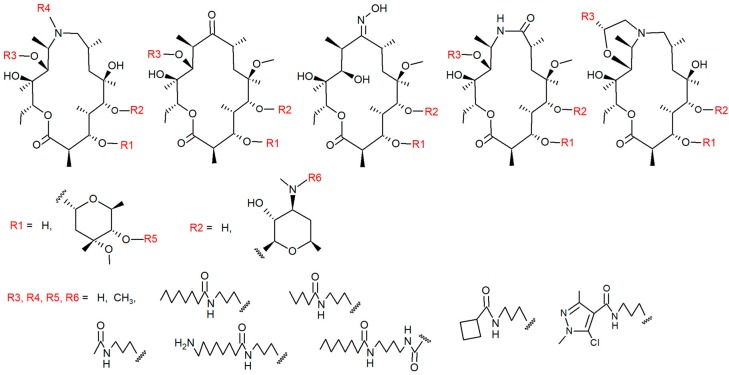
Generic structures of the rationally designed set of 69 macrocycles used in the project. The set was specifically created to cover a wide range of cellular accumulation intensities. Positions of various substituents R1–R6 in a 15- and 14-atom macrocycle are indicated in red [15,16].

Compound structures, distribution into the training and validation sets as well as their experimental data are provided in the Supplementary Materials. Generic structures of the used macrocycles are shown in Figure 1.

### 2.2. Prediction of Intracellular Accumulation from Molecular Structure

To enable the determination of intracellular accumulation only from the molecular structure, using our training set of 47 compounds, we developed three OPLS models as described in the Materials and Methods (Table 2). OPLS (Orthogonal projections to latent structures) is a projection method, and a modification to the traditional projections to latent structures (or partial least squares, PLS) method, where the aim is to separate the systematic variation in the descriptors into two parts: (1) one that is linearly related to the target variable (ACC Class), and (2) another that is orthogonal to the target variable.

Two of the three developed models predict ACC Class, based either on the calculated descriptors together with an experimental descriptor (LTR ACC Class) (Model 1), or based solely on calculated molecular descriptors (Model 2). Since the experimentally determined LTR ACC Class turned out to be a crucial descriptor in the successful prediction of accumulation (ACC Class), we developed the third OPLS model with the purpose to predict this experimental descriptor LTR ACC Class from the calculated molecular descriptors (Model 3). This way, we wanted to create an alternative, non-experimental way to obtain this crucial information for the prediction of intracellular accumulation (ACC Class).

**Figure 2:**
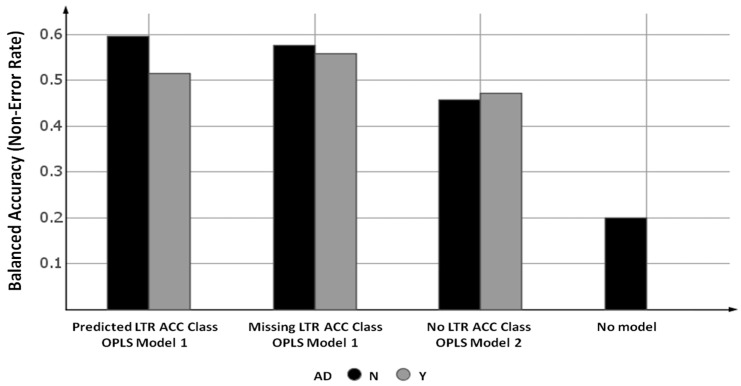
The balanced accuracies (non-error rates) of intracellular accumulation prediction (parameter ACC Class) for the validation set using two orthogonal projections to latent structures (OPLS) models (Models 1 and 2). Model 3 was also applied to predict the LTR ACC Class, and predicted values were used in Model 1 (denoted as the “Predicted LTR ACC Class”). Applicability domain (AD) considerations: AD = N (black), all compounds (30); AD = Y (grey), only compounds within AD (19).

To evaluate the developed prediction models, the validation set compounds were first predicted from the model based only on the 97 calculated physicochemical descriptors (OPLS Model 2), depicted in Figure 2 as the “No LTR ACC class”. The accuracies of the prediction of the ACC Class (five classes) for the validation set, taking the AD into account or not were 47.1 and 45.6%, respectively, which was more than 2-fold improvement in accuracy when compared to guessing the class by chance (“No model”).

### 2.3. Enrichment of the QSAR Model for Intracellular Accumulation with Experimental Imaging Data

The intracellular accumulation of the validation set compounds were further predicted using OPLS Model 1, which is based on 97 calculated and one experimental descriptor. In order to avoid the dependence on experimental data, this prediction was performed in two different ways: with and without the predicted LTR ACC Class data as the input descriptor. In the former case, the LTR ACC Class information was predicted using the OPLS Model 3 based on 97 calculated descriptors, while for the latter case, the LTR ACC Class descriptor was treated by OPLS as “missing data”. The results for these two sets of predictions are depicted in Figure 2 as the “Predicted LTR ACC Class” and “Missing LTR ACC Class”, respectively. The balanced accuracy of the model was slightly higher (60%) when using the predicted values for the LTR ACC Class as descriptor when compared to treating these values as missing (56% with applicability domain consideration). However, comparing these two sets of results using the McNemar’s Chi-squared test with continuity correction [18], there was no statistically significant difference between these two models (*p*-value = 0.134) at the 95% confidence level. 

**Figure 3:**
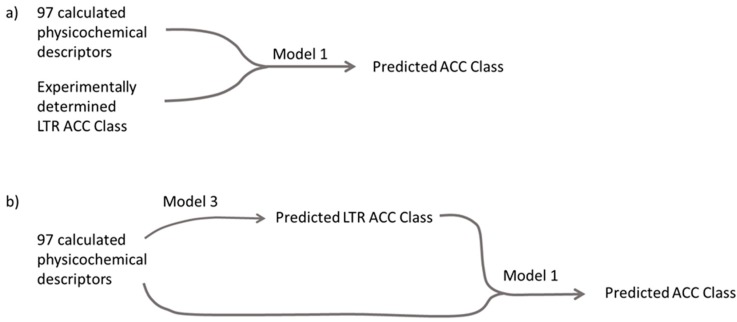
A schematic representation of developed prediction models for intracellular accumulation of compounds: (**a**) OPLS Model 1 developed with the training set; (**b**) Model 1 extended by Model 3 in the validation step. The model in (**b**) no longer requires experimental testing for the prediction of the ACC Class.

**Figure 4:**
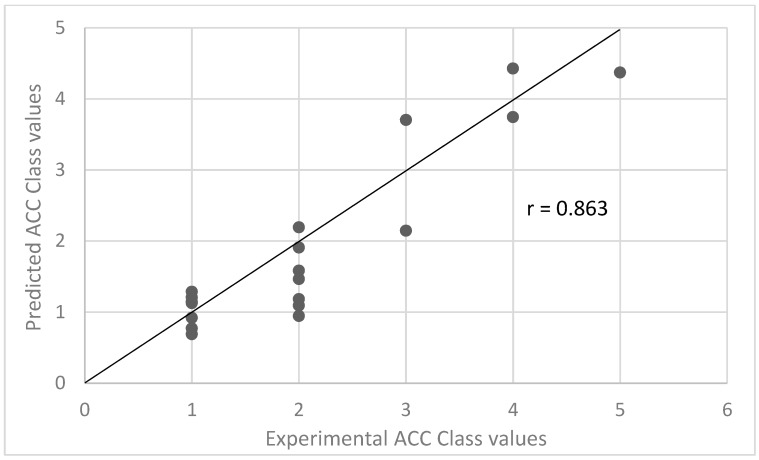
Correlation (Spearman correlation coefficient) between the experimental and predicted ACC Class as predicted by OPLS Model 1 using the LTR ACC Class values predicted by Model 3.

On the other hand, comparing the validation set prediction results from Model 2 with Model 1 by using the predicted LTR ACC Class as the descriptor (“No LTR ACC class” vs. “Predicted LTR ACC Class”), the difference between these two models was statistically significant (*p*-value = 0.023) at the 95% confidence level, according to the McNemar’s Chi-squared test. This indicates that the information provided by Model 3, developed for predicting the experimental parameter LTR ACC Class, is extremely useful for obtaining more accurate ACC Class predictions from Model 1. Since Model 3 is based only on calculated molecular descriptors, and it replaces the only experimental descriptor in Model 1, this information fusion resulted in a significantly improved Model 1, which now required only calculated structural descriptors (Figure 3). The balanced accuracy of this new, purely in silico descriptor-based model (“Predicted LTR ACC Class”) was 59.7%, which is an almost 3-fold improvement over guessing by chance (“No model”) with a high correlation between experimental and predicted ACC Class values with a Spearman coefficient of correlation R of 0.86 (Figure 4). Moreover, for the mispredicted compounds, the error in prediction was never larger than one class, which means that the prediction result was never far away from the actual experimentally determined values.

Accordingly, when treating LTR ACC Class values as “missing”, the benefits of the above described information fusion are omitted and the difference in validation set predictions from Model 1 (“Missing LTR ACC Class” in Figure 2) and from Model 2 (“No LTR ACC Class descriptor”) was not statistically significant at the 95% confidence level.

The derived OPLS Models 1–3 contained one predictive component for all three models and four, two, and three orthogonal descriptor (X) space components, respectively. The performance when randomly selecting 33% of the compounds in the training set as an “internal” validation set was similar when compared to the external validation set (BA: 0.46–0.60) with balanced accuracy (BA) values of between 0.43–0.66.

### 2.4. PLS Coefficients of the Two Models for the Prediction of Intracellular Accumulation

**Figure 5:**
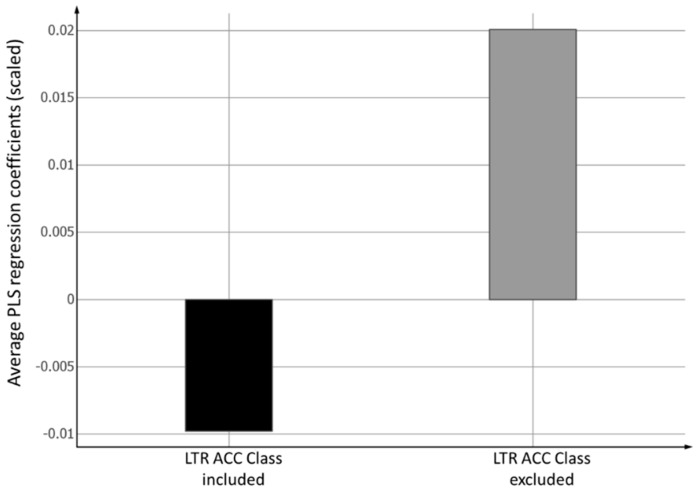
Average autoscaled PLS regression coefficients excluding the LTR ACC Class descriptor, with absolute values greater than 0.05 for Model 1 (LTR ACC Class included), and Model 2 (LTR ACC Class excluded), respectively.

**Figure 6:**
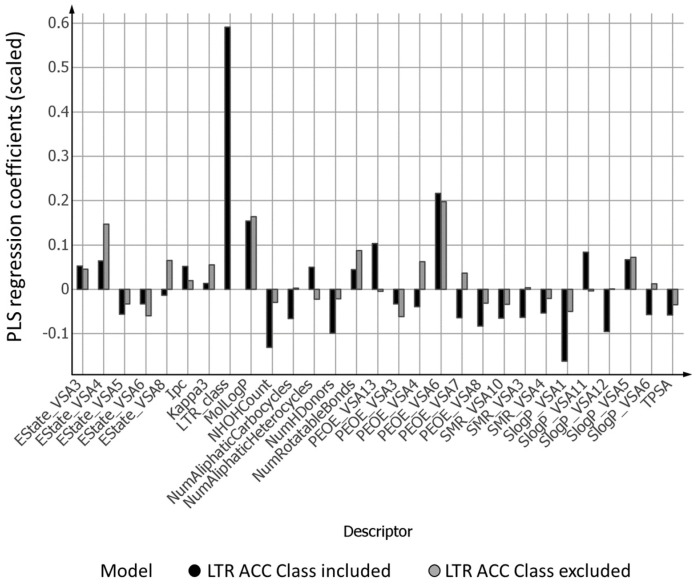
Autoscaled PLS regression coefficients with absolute values greater than 0.05 for Model 1 (LTR ACC Class included) and Model 2 (LTR ACC Class excluded).

The redistribution of information among the descriptors when including the experimental LTR ACC Class descriptor or not in the model is evident from Figure 5 and Figure 6. When excluding this single most important descriptor, the information carried by this variable must, as much as possible, be redistributed into the remaining descriptors. Since the LTR ACC Class descriptor has a positive value in Model 1, there is an average increase in values for the remaining physicochemical descriptors in Model 2 when compared to the corresponding ones in Model 1 (Figure 5). The same redistribution on an individual descriptor level is depicted in Figure 6. Most descriptors have a more positive coefficient in Model 2 when compared to Model 1. 

Inspection of the most important molecular descriptors from OPLS Model 1 (LTR_class, PEOE_VSA6, PEOE_VSA13, MolLogP, SlogP_VSA1, NHOHCount, NumHDonors) showed that they were related to various descriptions of partially charged van der Waals surface area contributions, lipophilicity, and hydrogen bonding, where the two first categories have a positive contribution with respect to accumulation, while the last category negatively influences the level of accumulation [19,20]. This is in line with previous observations that lipophilicity, hydrophobic regions, and the number of positive charges contribute positively to accumulation [15,16].

### 2.5. General Remarks

The aim of the present study was to develop purely in silico descriptor-based models for the prediction of intracellular accumulation of macrocyclic compounds, as measured by LC-MS/MS in NCI-H292 cells (ACC Class). To do that, we used calculated physicochemical molecular descriptors as well as experimental data on lysosomal volume change in response to test compounds (LTR ACC Class) determined by cell imaging, which was found previously to correlate extremely well with the intracellular accumulation measured by LC-MS/MS [17].

Experimental imaging data (LTR ACC Class) as a descriptor was found to have the highest PLS regression coefficient, indicating its strong contribution and high importance in the prediction of intracellular accumulation. In order to enable reliable predictions of accumulation even for compounds for which there are no experimental imaging data available, we developed a model for the prediction of imaging data from calculated molecular descriptors (OPLS Model 3). 

Using predicted imaging data from Model 3, together with the physicochemical descriptors in Model 1, was shown to have the highest balanced accuracy of all tested in silico models, and was performing statistically significantly better than the model based only on physicochemical descriptors. This means that the fusion of the information contained in Model 3 with Model 1 resulted in the improved model being capable of predicting new compounds without any experimental data.

It is noteworthy that the obtained in silico models were developed with calculated molecular descriptors based on 2D rather than a 3D structure, even for large molecules such as macrocycles. It is likely that most important information about the 3D structure is contained in the experimental LTR ACC Class descriptor. However, the remaining 2D molecular descriptors from Models 1 and 3 combined sufficiently well to describe the information necessary to obtain successful models. Moreover, the descriptors were relatively easy to calculate and are readily available, as they come from an open source software.

In our recent work, we have shown that the in vitro experimental 5-class model based only on the experimental imaging of lysosomal volume changes showed an extremely high accuracy of 81% in predicting the level of intracellular accumulation [17]. It is therefore not surprising that this descriptor turned out to be highly important in the here developed in silico models. We may suggest that for already synthesized compounds and compound libraries, the in vitro imaging model may be the easiest way to obtain a very accurate estimation of intracellular accumulation of compounds in high throughput format, provided that the facilities for this in vitro method are available. On the other hand, for yet non-existing compounds the purely in silico descriptor-based model (Figure 3b) with predicted imaging data offers, so far, the best way forward in predicting cellular accumulation and helping in the design of new macrocycle molecules with favorable cellular accumulation properties. This preselection would present a huge advantage for compounds with such a complex synthesis like macrocycles.

## 3. Materials and Methods 

### 3.1. Compounds

Test compounds included 69 macrocycles and eight standard non-macrocyclic drugs. Macrocycle synthesis and intracellular accumulation measured by LC-MS/MS in the NCI-H292 cell line in 3 h exposure was reported by Stepanic et al. (2011) [15] and Kostrun et al. (2017) [16]. The macrocyclic compounds used are represented by generic formulas in Figure 1.

The accumulation of eight standard non-macrocyclic drugs: acetaminophen (ACE), amiodarone (AMIO), amitriptyline (AMIT), chloroquine diphosphate (CHL), imipramine (IMI), indomethacin (IND), ofloxacin (OFL), and fluoxetine (FLU) was reported by Sanchez Garcia et al. (2018) [21].

The 77 compounds (69 macrocycles and eight non-macrocycles) covered the whole range of cellular accumulation intensities as reported for pharmaceuticals thus far, from no/low accumulation to extremely high. The training set consisted of 47 compounds (39 macrocycles and eight non-macrocycles) and the validation set contained 30 macrocycles. Compound structures as well as their distribution in both sets, together with their experimental data, can be found in the Supplementary Materials.

### 3.2. Physicochemical Descriptors

The investigated compounds were desalted and neutralized using Corina [22], followed by structure standardization using the IMI eTOX project standardizer (https://pypi.python.org/pypi/standardiser) in combination with the MolVS standardizer for tautomer standardization (https://pypi.python.org/pypi/MolVS).

A total of 97 different 2D physiochemical descriptors covering molecular size and shapes, charges, ring characteristics, lipophilicity, hydrogen bonding as well as various van der Waals surface area contributions of hydrophobic/hydrophilic, polarizability and electrostatic nature were calculated using RDKit (2017.09.01 release, https://www.rdkit.org): ‘Chi0’, ‘Chi0n’, ‘Chi0v’, ‘Chi1’, ‘Chi1n’, ‘Chi1v’, ‘Chi2n’, ‘Chi2v’, ‘Chi3n’, ‘Chi3v’, ‘Chi4n’, ‘Chi4v’, ‘EState_VSA1’, ‘EState_VSA10’, ‘EState_VSA11’, ‘EState_VSA2’, ‘EState_VSA3’, ‘EState_VSA4’, ‘EState_VSA5’, ‘EState_VSA6’, ‘EState_VSA7’, ‘EState_VSA8’, ‘EState_VSA9’, ‘FractionCSP3’, ‘HallKierAlpha’, ‘HeavyAtomCount’, ‘Ipc’, ‘Kappa1’, ‘Kappa2’, ‘Kappa3’, ‘LabuteASA’, ‘MolLogP’, ‘MolMR’, ‘MolWt’, ‘NHOHCount’, ‘NOCount’, ‘NumAliphaticCarbocycles’, ‘NumAliphaticHeterocycles’, ‘NumAliphaticRings’, ‘NumAromaticCarbocycles’, ‘NumAromaticHeterocycles’, ‘NumAromaticRings’, ‘NumHAcceptors’, ‘NumHDonors’, ‘NumHeteroatoms’, ‘NumRotatableBonds’, ‘NumSaturatedCarbocycles’, ‘NumSaturatedHeterocycles’, ‘NumSaturatedRings’, ‘PEOE_VSA1’, ‘PEOE_VSA10’, ‘PEOE_VSA11’, ‘PEOE_VSA12’, ‘PEOE_VSA13’, ‘PEOE_VSA14’, ‘PEOE_VSA2’, ‘PEOE_VSA3’, ‘PEOE_VSA4’, ‘PEOE_VSA5’, ‘PEOE_VSA6’, ‘PEOE_VSA7’, ‘PEOE_VSA8’, ‘PEOE_VSA9’, ‘RingCount’, ‘SMR_VSA1’, ‘SMR_VSA10’, ‘SMR_VSA2’, ‘SMR_VSA3’, ‘SMR_VSA4’, ‘SMR_VSA5’, ‘SMR_VSA6’, ‘SMR_VSA7’, ‘SMR_VSA8’, ‘SMR_VSA9’, ‘SlogP_VSA1’, ‘SlogP_VSA10’, ‘SlogP_VSA11’, ‘SlogP_VSA12’, ‘SlogP_VSA2’, ‘SlogP_VSA3’, ‘SlogP_VSA4’, ‘SlogP_VSA5’, ‘SlogP_VSA6’, ‘SlogP_VSA7’, ‘SlogP_VSA8’, ‘SlogP_VSA9’, ‘TPSA’, ‘VSA_EState1’, ‘VSA_EState10’, ‘VSA_EState2’, ‘VSA_EState3’, ‘VSA_EState4’, ‘VSA_EState5’, ‘VSA_EState6’, ‘VSA_EState7’, ‘VSA_EState8’, ‘VSA_EState9’. See Supporting Materials for more detail.

### 3.3. Experimental Descriptor LTR ACC Class—the Indirect High Throughput Experimental Measure of Cellular Accumulation

Imaging of the enlargement of lysosomal volume for 47 out of 77 compounds has been described in our previous study [17], where the lysosomal volume increase measured by fluorescent microscopy was found to highly correlate with the intracellular accumulation of compounds measured by LC-MS/MS. In that study, we developed a 5-class in vitro model for the prediction of cellular accumulation as determined by LC-MS/MS (ACC Class) from the changes in lysosomal volume. The obtained five classes of compounds according to the intensity of lysosomal volume enlargement were used as an experimental descriptor (referred to as LTR ACC Class) in the development of the in silico models for the prediction of intracellular accumulation in the present study. In this classification, class 1 denotes the lowest or no accumulation and class 5 represents an extremely high accumulation of a compound (Table 1).

### 3.4. Quantitative Structure-Activity Relationship Model (QSAR) Modeling

The dependent variable was experimentally determined intracellular accumulation measured by LC-MS/MS expressed as five classes (Table 1). The distribution of the test compounds in five classes by ACC Class and LTR ACC Class is shown.

The orthogonal PLS (OPLS) method (SIMCA 15 software package, Umetrics, Umea, Sweden) was used for data analysis [23]. The dataset was mean-centered and auto-scaled prior to analysis. Simca default parameter settings were used. OPLS, a modification of the traditional PLS method, aims to separate the systematic variation in the descriptor matrix into two parts: (1) linearly related to the target variable (ACC Class), and (2) orthogonal to the target variable, whereas the traditional PLS method only addresses part 1. Due to the rather low total number of available compounds, their distribution into the training and validation sets was guided by the availability of experimental data. Since the 47 compounds, for which both ACC Class and LTR ACC Class data were available, distributed well into five classes of the ACC Class, they were used as the training set. The remaining 30 compounds with ACC Class but no LTR ACC Class data were designated into the validation set. The training set of 47 compounds (with available ACC Class and LTR ACC Class data) was used to develop two different OPLS models for the prediction of intracellular accumulation determined by LC-MS/MS (Models 1 and 2) and one OPLS model for the prediction of intracellular accumulation class determined by lysosomal imaging (LTR ACC Class) (Model 3) (Table 2):

1. OPLS intracellular accumulation Model 1 using both the 97 physiochemical descriptors and the experimental LTR ACC Class information for 47 compounds with existing experimental LTR ACC Class information. The model was used to predict intracellular accumulation as measured in cells using LC-MS/MS.
2. OPLS intracellular accumulation Model 2 using only the 97 physiochemical descriptors for the same 47 compounds in Model 1. The model was used to predict intracellular accumulation as measured in cells using LC-MS/MS.
3. OPLS LTR ACC Class Model 3 using only the 97 physiochemical descriptors for 47 compounds with experimental LTR ACC Class information. The model was used to predict the lysosomal volume change as measured in cells using cell imaging (parameter LTR ACC Class), and which was previously found to correlate with intracellular accumulation determined by LC-MS/MS.

In order to validate the developed models, we used 30 compounds (validation set) with available ACC Class, but no LTR ACC Class data. To predict the ACC Class for the validation set, we used three different schemes:

- From OPLS Model 1 where the LTR ACC Class descriptor was treated as ‘missing’ data (“missing LTR ACC class”).
- From OPLS Model 1 where the predicted values from OPLS LTR ACC Class Model 3 were used as the LTR ACC Class descriptor (“predicted LTR ACC class”).
- From OPLS Model 2 (“no LTR ACC class”).

The applicability domains (AD) for the derived models were defined, by default, using the residual standard deviation (rsd) (i.e., remaining unused information in the data matrix), and the 95% confidence interval as the cut-off in relation to the average residual standard deviation in the descriptor matrix of the training set from 7-fold cross-validation (i.e., calculated using each of the left-out folds). If the rsd for the test compound was greater than two times the average rsd of the training set (~95% confidence interval), the test compound in question is regarded as out-of-AD.

Since an experimental model for predicting ACC Class already exists as the cell imaging method resulting in parameter LTR ACC Class, with the in silico models in this study, we specifically focused on validating models that would not need any experimental data for new compounds to be predicted. Therefore, all three schemes of validation are applicable for compounds where only the molecular structure is known.

Multi-class balanced accuracy (non-error rate) [24], which is not influenced by the imbalanced distribution of classes as well as Spearman rank correlation were used to evaluate the performance of the derived models.

## 4. Conclusions

In this study, we used the intracellular accumulation data of 69 macrocyclic compounds measured by LC-MS/MS to build QSAR models for the prediction of their intracellular accumulation. In addition to the molecular structure descriptors, we also used the experimental data of intracellular accumulation obtained by high throughput imaging of lysosomal volume changes. For compounds without any experimental data, our purely in silico model based on calculated molecular descriptors and predicted lysosomal imaging data, showed the most accurate classification predictions of five classes of intracellular accumulation reported so far, with a balanced accuracy of 60%. Bearing in mind the difficulty of purely in silico based predictions for such large molecules like macrocycles, and the importance of cellular accumulation in their pharmacology, the presented model can be of significant help in the design of the new macrocycle molecules with fine-tuned desired pharmacokinetic properties.

## Figures and Tables

**Table 1:** The five classes of compound accumulation levels in cells (ACC Class) and the distribution of test compounds into classes according to liquid chromatography coupled to tandem mass spectrometry—LC-MS/MS (ACC Class) or the lysosome volume change measurements (LTR ACC Class).

ACC Class	I/E	% AZI	Accumulation	No of cpds Per class ^a^	No of cpds Per Class ^b^
**1**	0–7	0–14%	No/low	22	14
**2**	7.5–33	15–66%	Moderate	23	10
**3**	33.5–92	67–184%	High	14	7
**4**	92.5–220	185–440%	Very high	9	11
**5**	>220	>440%	Extremely high	7	5

^a^ according to the ACC Class, ^b^ according to the LTR ACC Class. I/E = accumulation (ACC) expressed as intracellular to extracellular concentration ratio measured by LC-MS/MS, % AZI = accumulation (ACC) expressed relative to control compound azithromycin [17].

**Table 2:** An overview of the orthogonal projections to latent structures (OPLS) models developed with the training set.

OPLS Model	Predicted Endpoint	Name of the Dependent (Predicted) Parameter	No. of Classes of the Dependent (Predicted) Parameter	Descriptors Used in the Model ^a^
Model 1	Intracellular accumulation measured by LC-MS/MS	ACC Class	5	● 97 calculated physicochemical descriptors ● Experimentally measured LTR ACC Class
Model 2	Intracellular accumulation measured by LC-MS/MS	ACC Class	5	● 97 calculated physicochemical descriptors
Model 3	Intracellular accumulation measured by cell imaging	LTR ACC Class	5	● 97 calculated physicochemical descriptors

^a^ See Section 3.2 and Supporting Materials for a detailed description.

## Supplementary Materials

Supplementary Materials can be found at https://www.mdpi.com/1422-0067/20/23/5938/s1.

## Abbreviations

ACC	Accumulation of compounds in cells expressed as either I/E or % of the accumulation of the reference compound azithromycin
ACC Class	Class (level) of accumulation (determined from LC-MS/MS data on ACC)
AD	Applicability domain
ADME	Absorption distribution metabolism and excretion
AZI	Azithromycin, a macrocycle antibiotic
CAD	Cationic amphiphilic drug
I/E	Intracellular to extracellular concentration ratio; a measure of accumulation
LC-MS/MS	Liquid chromatography coupled to tandem mass spectrometry
LTR ACC Class	Class (level) of accumulation (determined by an indirect method of cell imaging of lysosome volume change)
OPLS	Orthogonal projections to latent structures
QSAR	Quantitative structure-activity relationship model
